# Influence of Different Drying Techniques on Phenolic Compounds, Antioxidant Capacity and Colour of *Ziziphus jujube* Mill. Fruits

**DOI:** 10.3390/molecules24132361

**Published:** 2019-06-26

**Authors:** Aneta Wojdyło, Krzysztof Lech, Paulina Nowicka, Francisca Hernandez, Adam Figiel, Angel Antonio Carbonell-Barrachina

**Affiliations:** 1 Department of Fruit, Vegetable and Plant Nutraceutical Technology, Wrocław University of Environmental and Life Sciences, 37 Chełmońskiego Street, 51-630 Wroclaw, Poland; 2 Institute of Agricultural Engineering, Wrocław University of Environmental and Life Sciences, 37/41 Chełmońskiego Street, 51 630 Wrocław, Poland; 3 Department of Plant Sciences and Microbiology, Research Group “Plant Production and Technology”, Escuela Politécnica Superior de Orihuela, Universidad Miguel Hernández de Elche, Ctra. de Beniel, km 3.2, 03312 Orihuela, Alicante, Spain; 4 Department of Agro-Food Technology, Research Group “Food Quality and Safety”, Escuela Politécnica Superior de Orihuela (EPSO), Universidad Miguel Hernández de Elche, Ctra. de Beniel, km 3.2, 03312 Orihuela, Alicante, Spain

**Keywords:** jujube, microwave power levels, air temperature, bioactive compounds

## Abstract

This study was to present the effect of different parameters of combined methods of drying such as vacuum-microwave (VMD: 480, 120 W), hot air (CDD: 70, 60, 50 °C) and combined methods as pre-drying by CD and finish drying by VMD (CD-VMD: 60 °C + 480/120W) in order to avoid a rapid increase in temperature at the critical moisture content of ca. 1 kg/kg dm (dry mass). Control samples were prepared by freeze-drying (FD). Drying kinetics, including the temperature profile of dried material, as well as on some quality factors of the finished product as phenolic compounds, antioxidant capacity, and color were evaluated. The increase in air temperature during CD as well as the increase in material temperature during VMD deteriorated dried product quality in terms of the content of phenolic compounds, antioxidant activity and color. Dried jujube fruits have a long shelf life and therefore may be a fine alternative to fresh fruit all year round.

## 1. Introduction

Nowadays dried fruits and vegetablesare highly popular valuable healthy snacks. Drying affects the fruit appearance and chemical composition but it allows for effective handling of raw materials and prolonging their shelf life as it inhibits enzymatic degradation and limits microbial growth [1].

Selection of an adequate drying method and its parameters yields a product with high antioxidant activity, only slightly changed in appearance as compared to fresh fruit, and with a more favorable taste. Considering consumer preferences, appropriate drying method should be selected, so as to retain maximum levels of bioactive compounds in the final product. Dried fruit snacks are good sources of dietary fibre, minerals, vitamins, and bioactive compounds. Their antioxidant properties are due mainly to the presence of carotenoids, phytosterols, phenolic compounds and vitamins C and E [2]. 

Many reports claim that choosing right parameters of the drying method is as important as choosing the method itself [3,4,5,6]. Currently, one of the most popular drying metod is microwave drying, because contrary to hot air drying method, it reduces the drying time of plant materials without any meaningful decline of quality. At an industrial level, food processing using this technique has been reported to be both cost effective and feasible [7]. Systems of drying combining (such as: microwave and hot air drying) not only increase drying rates but also responsible for quality of the dry products [8,9,10]. Nowadays, day by day microwave drying techniques is widely used in combination with pre-drying by hot air-drying systems which removes free water from the product surface, and finished by microwave when energy from microwave removes water from inside the product [6,11]. 

Jujube (*Ziziphus jujuba* Mill.) fruits are highly favored by consumers, as they are tasty and rich in nutrients, especially vitamins, minerals, and polyphenols [12,13,14]. The fruits are a good source of natural antioxidant compounds, namely polyphenols that confer numerous health benefits i.e. show antiobesity antiproliferative, antitumor, antioxidant, antiinflamatory, and proapoptopic properties, and may protect against cardiovascular diseases and type II diabetes [15]. Generally, jujube fruits are eaten fresh, however, their shelf-life is short (2–4 days at ambient temperature) and their rapid decay is problematic for postharvest management and advance processing [16,17].

Therefore, the aim of this present study was to determine the effects of different drying methods, such as vacuum-microwave drying (VMD) at different powers (120 and 480 W), hot air drying (CD) at different temperatures (50, 60, 70 °C), and hot air pre-drying followed by vacuum-microwave finish drying (CD-VMD) on the quality of three different jujube cultivars (‘GAL’, ‘MSI’, and ‘PSI’). In obtain sample it was evaluated drying kinetics, including temperature profile of the dried material, and the quality of the dried products, including color, total phenolic compounds (TPC), and antioxidant capacity (ORAC). Finally, freeze drying (FD) was used as a reference or control method, as it provides high quality of the final products.

## 2. Results and Discussion

### 2.1. Drying Kinetics

**Figure 1:**
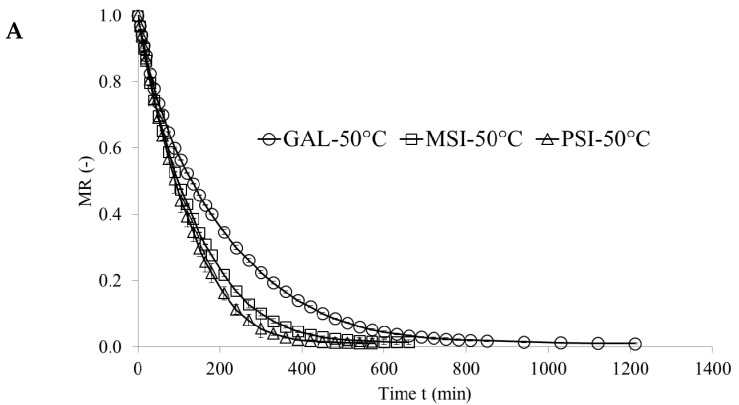

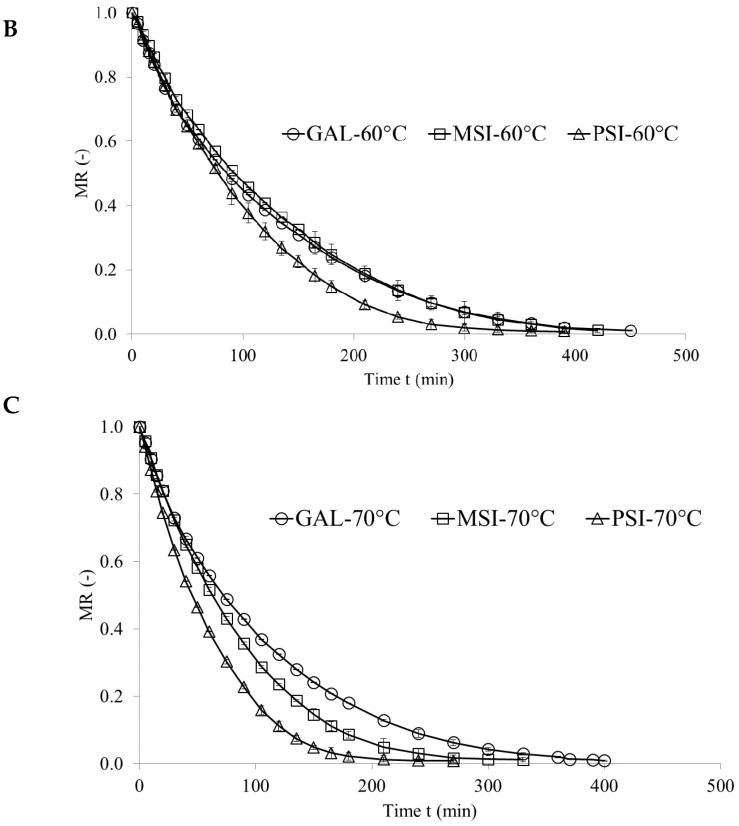
(**A**) Drying kinetics of jujube fruits during CD 50 °C; (**B**) Drying kinetics of jujube fruits during CD 60 °C; (**C)** Drying kinetics of jujube fruits during CD 70 °C.

**Figure 2:**
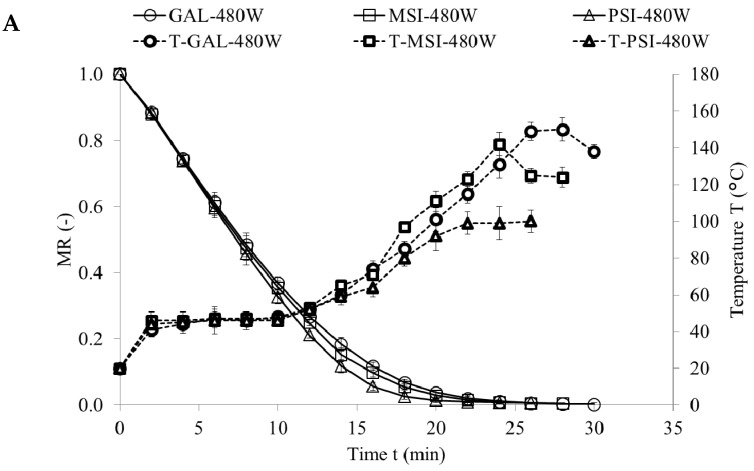

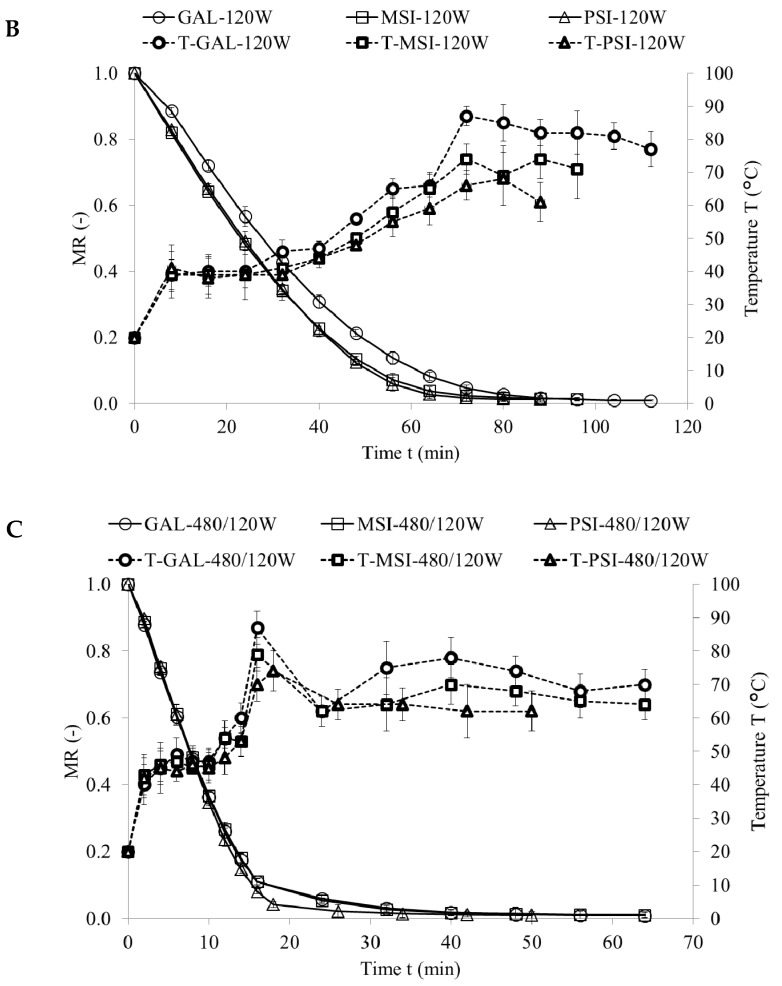
(**A**) Drying kinetics of jujube fruits during VMD 480 W; (**B**) Drying kinetics of jujube fruits during VMD 120 W; (**C**) Drying kinetics of jujube fruits during VMD 480/120 W.

**Figure 3:**
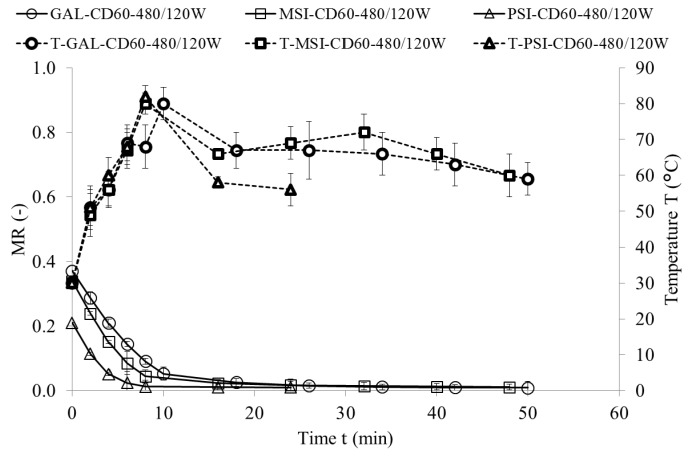
Drying kinetics of jujube fruits during pre-drying by CD (60 °C) and finished by VMD (480/120W).

Figure 1, Figure 2 and Figure 3 show drying kinetics of jujube fruits as a function of MR change over time. Preliminary tests identified modified Page model as the best one describing the drying kinetics (Equation (1)):

(1) $$MR=A\cdot e^{-k\cdot t^{n}}$$

where *A, n, k* and *t* are constants and drying time, respectively.

This model was used before to characterize the drying kinetics in jujube [16], chokeberry [17] and plum [18]. Table 1 presents the model constants, coefficients of determinations (*R*^2^), root mean square error (RMSE), maximum temperature and final moisture content and drying time. The values of RMSE below 0.0181 and of *R*^2^ above 0.9879 demonstrate very good fit of the model to the empirical data [19]. Parameter A represents MR value at the beginning of the drying, which equals 1 for CD and VMD. For CD-VMD method this variable reaches MR after hot air pre-drying. Similar results were obtained for drying of pomegranate arils [20]. Parameters *n* and *k* determine the drying rate—the greater they are, the shorter the drying time [17]. In all cultivars they were significantly higher in VMD variant than in CD. Materials with high water content heat up faster during microwave drying as they absorb more microwave power. This considerably improves the drying time [21]. Additionally, reducing pressure increases the pressure gradient and makes the drying process up to five times faster [7]. The shortest drying time (26 min) was achieved for cv. ‘PSI’ dried by VMD at 480 W, and the longest (1210 min) for cv. ‘GAL’ exposed to hot air drying at 50 °C. ‘PSI’ fruits featured the highest initial moisture content (4.72 kg·kg^−1^ d.w.), but their drying time was shorter and maximum sample temperature during VMD was lower than in other cultivars (‘GAL’ and ‘MSI’). This might be due to how water is bound by cellular layout and structure in individual cultivars (cv. ‘PSI’ had higher cell density than ‘GAL’, data not present), and to plant defense mechanisms against water loss [22]. Final moisture content of dried fruits reported by [16] was below 5.66%, and this corroborated our results for jujube fruits (Table 1.)

### 2.2. Energy Consumption

**Figure 4:**
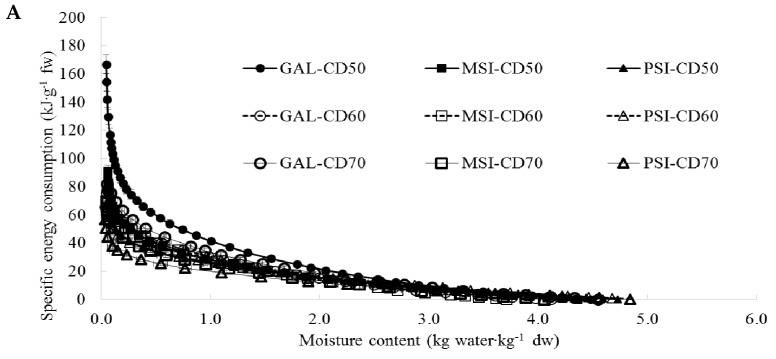

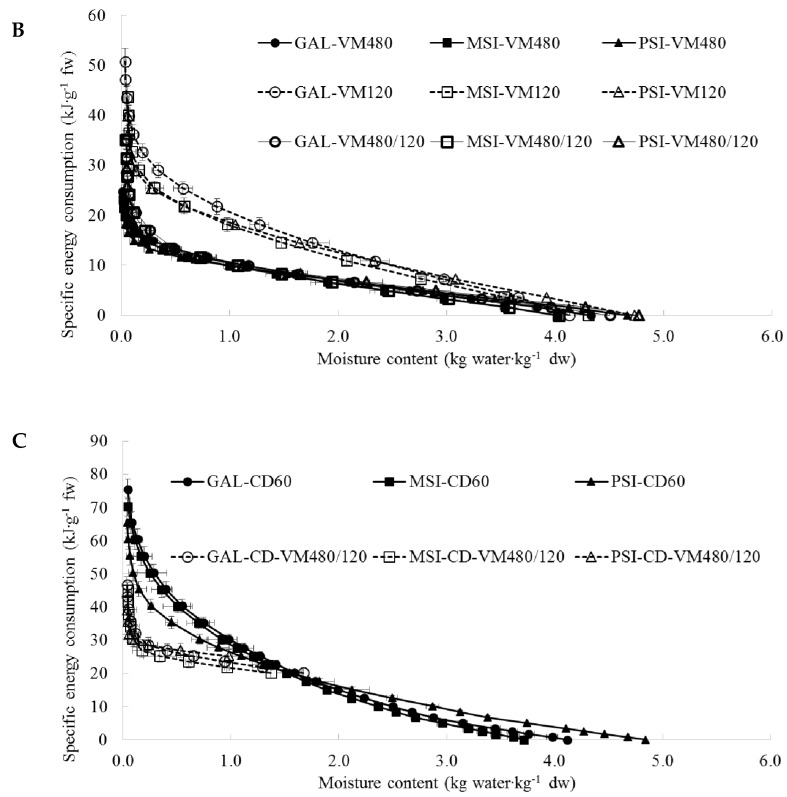
(**A**) Profiles of specific energy consumption during CD of jujube fruits. (**B)** Profiles of specific energy consumption during VMD of jujube fruits. (**C**) Profiles of specific energy consumption during CD 60 °C and CD-VMFD (60 °C 480/120W) of jujube fruits.

Figure 4A–C show specific energy consumption profiles depending on material moisture content during CD (Figure 4A), VMD (Figure 4B) and CD-VMD (Figure 4C). The specific energy consumption is expressed in kJ·g^−1^ fresh weight (f.w.). We observed a rapid growth in energy consumption in materials with low moisture content (below 0.25 kg·kg^−1^ d.w.), that is at the end of the drying, when the process slows down as water is removed from inside the material (internal diffusion). Similar energy consumption profiles were reported for garlic [9] and pomegranate [8]. Table 1 presents total specific energy consumption expressed in kJ·g^−1^ f.w. and kJ·g^−1^ water. The variable was the lowest during VMD at 480 W (21.5 kJ·g^−1^ f.w., 26.27 kJ·g^−1^ water) for cv. ‘PSI’, and the highest during CD at 50 °C (166.56 kJ·g^−1^ f.w., 205.77 kJ·g^−1^ water) for cv. ‘GAL’. Increased air temperature during CD and higher microwave power during VMD resulted in lower energy consumption. Similar conclusions were drawn following hot air drying of pomegranate fruits [23] and microwave drying of parsley leaves [24]. Combined drying (CD-VMD) reduced energy consumption by over 1.5 times as compared with CD. The same was reported by Jiang et al. [25] who experimented with drying okra. 

**Figure 5:**
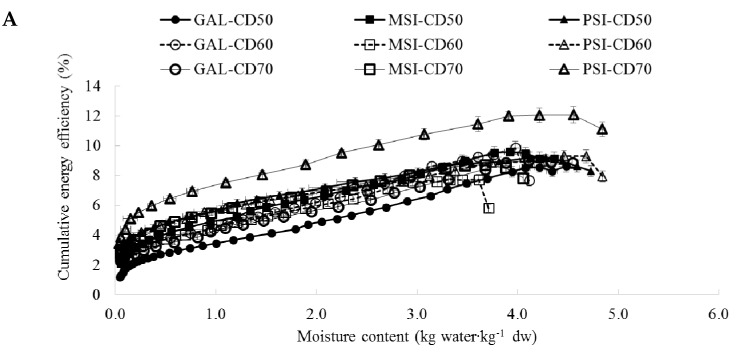

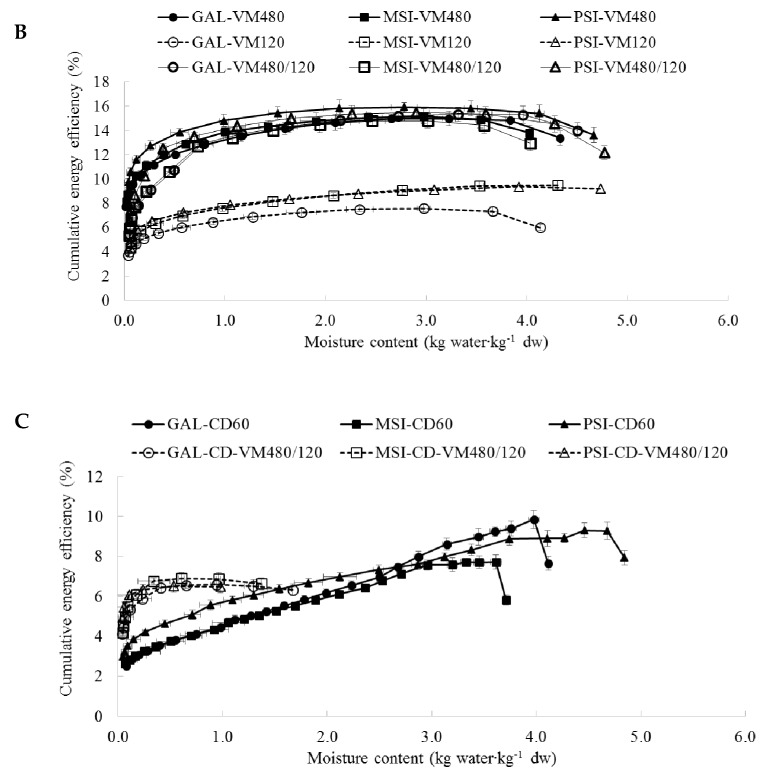
(**A**) Cumulative energy efficiency during CD of jujube fruits. (**B**) Cumulative energy efficiency during VMD of jujube fruits. (**C**) Cumulative energy efficiency during CD 60 °C and CD-VMD (60 °C 480/120W) of jujube fruits.

Figure 5 shows cumulative energy efficiency profiles. The cumulative energy efficiency closely correlates with specific energy consumption—increased energy consumption considerably reduces drying efficiency [26]. Table 1 displays final cumulative energy efficiency. It was the highest during VMD at 480 W (8.98%) for cv. ‘PSI’ and the lowest during CD at 50 °C (1.16%) for cv. ‘GAL’.

### 2.3. Color

Table 2 shows color parameters for the flesh of fresh and dried jujube fruits. L*, a* and b* were highly similar in the fresh material for all three cultivars. Irrespective of drying method, L* was lower in dried than fresh fruits, which means the fruits darkened. Chen et al. [16] reported an increase in L* in jujube fruits after drying but the value of this variable in fresh fruits was nearly two times lower than in our study. Such considerable differences may be due to not only different measurement methods but also to the flesh color that is a cultivar specific feature. Changes in a* and b* variables were more strongly affected by the drying method, and were greater in fruits exposed to VMD than CD. Table 2 shows total color change range (*d*E*) as compared with fresh fruits. *d*E* was the lowest in FD samples, and the highest in the material dried in the microwave at high microwave power (480 W). The changes are associated with high sample temperature (Table 1) that causes formation of brown compounds in Maillard reaction [23]. Color changes were less pronounced in cv. ‘PSI’ than in ‘GAL’ and ‘MSI’, which was probably due to shorter drying time and lower sample temperature. 

### 2.4. Total Phenolic Compounds and Antioxidant Activity

Control or reference values of total phenolic content (TPC) in the freeze-dried (FD) fruits were 3048, 3404, and 4454 mg/100 g d.w. for ‘GAL’, ‘MSI’, and ‘PSI’, respectively (Table 3). Similar initial values were reported by other researchers [16,27]. The main phenolic compounds in jujube fruits were flavan-3-ols (99% of total polyphenolic compounds in ‘GAL’, 97% in ‘PSI’, and 95% in ‘MSI’), with polymeric proanthocyanidins (PP) predominating and flavonols being the less abundant group. 

Drying method (FD, CD, VMD, and CD-VMD) and drying conditions significantly affected the contents of polyphenolic compounds. TPC content in all dried jujube samples followed the order FD >> VMD ≥ CD-VMD > CD. In terms of retaining TPC content the most efficient drying methods were FD > VMD at 480/120 W > CD-VMD (50 °C and 480/120W) > VMD at 120W > VMD at 480 W > CD at 50 °C. However, differences between the methods are not significant (*p* > 0.05). The study clearly indicates that TPC contents are retained more effectively (*p* < 0.05) when drying involves combined methods, such as pre-drying by CD and finished by VMD (CD-VMD) or even VMD with power adjustment along moisture content reduction, than the traditional hot-air drying (especially at 60 and 70 °C). 

We noticed that an increase in VMD power from 120 to 480 W reduced TPC in jujube fruits from all three cultivars (Table 3) but the trend was not significant (*p* > 0.05). Therefore, reducing the microwave power during VMD (from 480 W to 120 W) to avoid sample overheating resulted in significantly higher content of TPC. This trend was confirmed for ‘GAL’ and ‘MSI’. 

As expected, the biggest changes in TPC were observed in CD samples that showed a clear dependency between the hot air temperature and polyphenol loss in all jujube cultivars (the higher the temperature—the lower TPC). Convective drying at 50 °C allowed for retaining maximum TPC.

Microwave heating inactivates degrading enzymes much faster than convective heating [4], yet a loss of phenolic compounds was measured. Gao et al. [4,12] showed that oven heating at 70 °C rapidly inactivates polyphenol oxidases in jujube fruits. However, the enzymes may be active even earlier and degrade phenolic compounds at the initial stages of drying. Microwave-drying caused an insignificant (5%) drop in phenolic compounds in jujube fruits [4].

Chen et al. [16] suggested that temperature is more important than time in drying of jujube fruits. They showed that an increase in the drying temperature, from 70 to 80 °C, significantly reduced TPC. Similar findings were reported for vacuum-dried aronia fruits [5]. The authors of the study found that combining microwave and vacuum drying and reducing the power or wattage of the microwaves at the final stage of the process may significantly reduce the product temperature and limit the loss of bioactive compounds, thus improving the product quality [5]. As a consequence, and theoretically, VMD should yield products with higher content of nutrients and aroma compounds than CD.

Antioxidant activity (Table 3) of jujube fruits, similarly as polyphenols content, were related by the drying methods. The highest values (*p* < 0.05) of ORAC were found in dried fruits of cvs. ‘PSI’ > ‘MSI’ > ‘GAL’, with values of 60.63, 52.73, 43.30 mmol TE/100 g d.w., respectively. As prospective, the antioxidant activity was the highest in FD samples (72.45, 66.67 and 48.56 mmol TE/100 g d.w., for cv. ’MSI’, ‘PSI’ and ‘GAL’, respectively). VMD was the second most effective method at retaining such bioactive compounds as polyphenols and ORAC value, especially sample treated by at 120W. Considering the microwave power, the smallest loss was observed for combined (CD-VMD) method and power reduction from 480 W to 120 W. High air temperature (especially 60 or 70 °C) during dehydration process caused significantly (*p* < 0.05) degradation of biologically active compounds that may also exhibit antioxidant properties. Similarly, Wojdyło et al. [28] showed the greatest reduction of antioxidant activity at 70 °C (48%) while drying sour cherry fruits. As a conclusion, a high temperature causes faster degradation of the compounds responsible for the antioxidant activity. 

## 3. Materials and Methods 

### 3.1. Reagents and Standards

(-)-Epicatechin, (+)-catechin, quercetin, and kaempferol -3-*O*-glucoside and -3-*O*-rutinoside were purchased from Extrasynthese (Lyon, France). Ascorbic acid, trolox, phloroglucinol, acetonitrile and methanol for UPLC were purchased from Sigma-Aldrich (Steinheim, Germany). 

### 3.2. Plant Material and Sample Preparation

Approximately 2 kg of jujube fruits (*Z. jujube*) from each cvs. as ‘*GAL*’, ‘*MSI*’, ‘*PSI*’, were manually hand harvested from 20-year-old trees (from 3 trees cultivars) a farm in the village of San Isidro province of Alicante, Spain (19 m above sea level; 38°10’22, 29’’ N × 0°51’36,138’’ W); Jujube fruits before drying were pitted and cut for pieces.

### 3.3. Drying Experiments

Jujube samples approx. 60 g, were subjected to four different drying methods, which was continued until the moisture content of samples equaled 0.05 kg/kg dm:

(i) Hot air drying (CD) was conducted using dryer designed and built at the Institute of Agricultural Engineering (Wrocław University of Environmental and Life Sciences, Wrocław, Poland) [3]. Air velocity was 1 m/s and hot air temperatures during process were 50, 60 and 70 °C.
(ii) Vacuum-microwave drying (VMD) was carried out in dryer SM-200 (Plazmatronika S.A., Wroclaw, Poland) [3]. During VMD the microwave power was set to at 120 W, 480 W and 480/120 W (Microwave power was reduced to 120 W at the initial microwave power of 480 W, when the maximum temperature of sample was higher than 75 °C). The pressure in the VMD chamber varied between 4 and 6 kPa.
(iii) Combined drying (CD-VMFD) consisted of hot-air pre-drying (CD) at a temperature of 60 °C, followed by VMFD at 480/120 W, the hot-air pre-drying time was 120 min.
(iv) Freeze-drying (FD) was used as the control sample carried out used the dryer Alpha 1-4 LSC (Martin Christ GmbH, Osterode am Harz, Germany) during 24 h. During FD the pressure was reduced to 0.960 kPa. The temperature of shelves and drying chamber were 26 and -60 °C, respectively.

### 3.4. Drying Confirmed Kinetics

According to sample mass losses measured during drying was evaluated drying kinetics for convection and vacuum drying methods. The moisture ratio *MR* was determined using the following equation [29]:

(2) $$MR=\frac{M}{M_{0}}$$

where *M* is the actual moisture content and *M*_0_ is the initial moisture content.

The initial moisture contents of fresh jujube were 4.32, 4.12, and 4.72 kg/kg dry matter (dm) for ‘GAL’, ‘MSI’, and ‘PSI’, respectively.

The moisture content of dried samples was determined by drying the previously ground samples in a vacuum dryer (SPT-200, ZEAMiL Horyzont, Krakow, Poland) for 24 h at temperature 80 °C and pressure 300 Pa.

### 3.5. Energy Consumption 

The energy consumption during drying was calculated according to [9]. The energy efficiencies for CD, VMD and CD-VMD were determined as the ratio of energy necessary for evaporation of free water from the sample to the energy consumed while drying. The specific energy consumptions for CD, VMD and CD-VMD were determined as the ratio of energy consumption to the initial mass of the sample expressed as kJ·g^−1^ fw or as the ratio of energy consumption to the mass of water removed from the sample during drying expressed as kJ·g^−1^ water.

### 3.6. Colour 

The colour was determined on the surface of samples from the flesh side with reference to the colour space, CIE L*a*b* system using a Minolta Chroma Meter CR-400 (Minolta Co., Ltd., Osaka, Japan). The total change in the colour (dE) was calculated following the equation as described by [30]. The measurements were done in five replicates. 

### 3.7. Determination of Total Phenolic Compounds (TPC) by UPLC-PDA-FL Method

A sample for the analysis of polyphenols was prepared as described previously by Wojdyło et al. [20]. The sample for quantitative (UPLC-PDA-FL; Waters, Milford and Taunton, Massachusetts, USA) analysis of total polyphenols expresses as sum of flavonols (as sum of quercetin and keampferol derivatives)) and flavan-3-ols (as sum of monomers, dimers, polymeric procyanidins) were performed as described previously by Wojdyło et al. [14]. Prior to the measurements, the equipment was calibrated using a standard for flavonol compounds were used quercetin-3-*O*-glucoside (at 0,1 to 5 mg), and for flavan-3-ols were used (-)-epicatechin (at 0.1 to 5 mg). All measurements were repeated three times, and expressed as mean value as mg/100g dm. 

### 3.8. Determination of Antioxidant Activity 

The extraction of sample for the antioxidant analysis was prepared as described previously by Wojdyło et al. [20]. The ORAC assay was determined as previously described by Ou et al. [21] using a RF-5301 PC spectrofluorometer (Shimadzu, Kyoto, Japan). Results were expressed as mmol TE/100g dm.

### 3.9. Statistical Analysis

An ANOVA was performed using Statistica version 12.0 (StatSoft; Krakow, Poland), and means were separated by Duncan’s multiple range test. All analyzes were performed duplicated and present as mean value ± standard deviation. TableCurve 2D Windows v 5.01 (Jandel Scientific Software, San Jose, CA, USA) enabled mathematical modelling with the highest values of determination coefficient (*R*^2^) and the lowest values of root-mean-square error (RMSE).

## 4. Conclusions

The study identified fruits of cv. ‘PSI’ as the most appropriate for drying, despite the highest initial moisture content. Drying of this cultivar was the most efficient due to the shortest drying time and the lowest energy consumption. Furthermore, samples of cv. ‘PSI’ reached satisfactory level of dryness at the lowest temperature, which most effectively limited the loss of polyphenolic compounds and retained high antioxidant activity. ‘PSI’ fruits experienced also the smallest change in color (*d*E*).

Hot air drying at low temperature (CD 50 °C) was the best method (except for control FD), considering the content of polyphenols, antioxidant activity and color parameters. However, it required a few times more energy than microwave and vacuum drying. VMD method, particularly at high power values (480 W) heated the samples to high temperature that adversely affected fruit color (browning) and degraded polyphenolic compounds. Therefore, the combined drying method seems to be the most effective, as it provides good quality dried jujube fruits and requires relatively low energy consumption.

## Figures and Tables

**Table 1:** Drying time, maximum temperature of the samples, final moisture content, final specific energy consumption, cumulative energy efficiency and constants of the models describing the drying kinetics of jujube fruits.

Cultivars	Drying Conditions	Constants			Statistics		Drying Time (min)		Tmax (°C)	Mcwb (%)	Final Specific Energy Consumption		Cumulative Energy Efficiency (%)
		*A*	*k*	*n*	RMSE	*R* ^2^	CD	VMD			kJ·g^−1^ fw	kJ·g^−1^ water	
‘GAL’	VMD 480	1	0.0345	1.481	0.0133	0.9984	-	30	150 ± 7j *	0.95	24.76 ± 1.29ab	30.54 ± 1.59ab	7.73 ± 0.22k
	VMD 120	1	0.0054	1.465	0.007	0.9996	-	112	87 ± 3f	3.54	50.74 ± 2.64f	63.58 ± 3.31f	3.71 ± 0.13f
	VMD 480/120	1	0.0436	1.379	0.0167	0.9975	-	64	87 ± 5fg	4.04	35.13 ± 1.83c	43.34 ± 2.25c	5.45 ± 0.22i
	CD-VMD 480/120	0.372	0.111	1.212	0.0103	0.9924	120	50	80 ± 5defg	3.75	46.68 ± 2.43ef	57.49 ± 2.99e	4.10 ± 0.13g
	CD 50 °C	1	0.0076	0.930	0.008	0.9994	1210	-	50 ± 2a	4.24	166.56 ± 6.66k	205.77 ± 8.23l	1.16 ± 0.04a
	CD 60 °C	1	0.0085	0.994	0.0137	0.9982	420	-	60 ± 2b	4.30	75.52 ± 3.02i	94.92 ± 3.8i	2.51 ± 0.08cd
	CD 70 °C	1	0.0102	0.989	0.0093	0.9992	450	-	70 ± 2c	3.76	65.52 ± 3.28h	103.32 ± 4.65j	2.30 ± 0.07bc
‘MSI’	VMD 480	1	0.0342	1.506	0.0151	0.9979	-	28	142 ± 7i	1.78	23.12 ± 1.2a	29.00 ± 1.51a	8.14 ± 0.28l
	VMD 120	1	0.0098	1.368	0.0137	0.9982	-	96	74 ± 5cde	5.64	43.65 ± 2.27de	54.55 ± 2.84de	4.33 ± 0.14g
	VMD 480/120	1	0.0404	1.406	0.0154	0.9979	-	64	79 ± 5defg	4.42	35.02 ± 1.82c	44.21 ± 2.3c	5.34 ± 0.21i
	CD-VMD 480/120	0.341	0.154	1.216	0.0127	0.9957	120	48	80 ± 3defg	4.20	45.02 ± 2.34e	56.67 ± 2.95e	4.16 ± 0.13g
	CD 50 °C	1	0.0064	1.025	0.007	0.9996	660	-	50 ± 2a	5.53	91.08 ± 4.1j	113.48 ± 5.11k	2.10 ± 0.1b
	CD 60 °C	1	0.0057	1.066	0.0131	0.9984	420	-	60 ± 2b	4.20	70.28 ± 2.81h	90.29 ± 3.61hi	2.64 ± 0.08d
	CD 70 °C	1	0.0067	1.126	0.0088	0.9993	330	-	70 ± 2c	4.56	69.06 ± 3.45h	87.12 ± 4.36h	2.73 ± 0.1de
‘PSI’	VMD 480	1	0.0295	1.604	0.0181	0.9972	-	26	100 ± 6h	2.74	21.50 ± 1.12a	26.27 ± 1.37a	8.98 ± 0.3m
	VMD 120	1	0.0077	1.440	0.0171	0.9973	-	88	68 ± 8c	5.66	39.84 ± 2.07cd	48.89 ± 2.54cd	4.83 ± 0.15h
	VMD 480/120	1	0.0302	1.563	0.0146	0.9981	-	50	74 ± 6cd	4.84	29.46 ± 1.53b	36.02 ± 1.87b	6.55 ± 0.3j
	CD-VMD 480/120	0.21	0.285	1.136	0.0072	0.9879	120	24	82 ± 3efg	4.17	39.13 ± 2.93cd	47.66 ± 3.57c	4.95 ± 0.17h
	CD 50 °C	1	0.0045	1.121	0.0073	0.9996	570	-	50 ± 2a	4.76	78.58 ± 3.54i	96.22 ± 4.33i	2.47 ± 0.08cd
	CD 60 °C	1	0.005	1.141	0.0119	0.9988	390	-	60 ± 2b	3.85	65.52 ± 3.28h	79.71 ± 3.99g	2.99 ± 0.07e
	CD 70 °C	1	0.0094	1.133	0.0105	0.9991	270	-	70 ± 2c	3.60	56.73 ± 2.84g	68.98 ± 3.45f	3.45 ± 0.09f

* Values followed by the same letter ± standard deviation; within the same column, are not significantly different (*p* < 0.05, Duncan’s multiple range test); FD-freeze drying; CD-convective drying; VMD-vacuum-microwave drying; CD-VMD- convective-vacuum-microwave drying; A, k and n are constants of the modified Page model; RMSE-mean square errors; *R*^2^-determination coefficient; Tmax-temperature maximal; Mcwb-moisture content wet basis; in each column different letters mean significant differences between samples.

**Table 2:** Colour parameters as affected by different drying methods of jujube fruits.

Cultivars	Drying Conditions	*d*E*	L*	a*	b*
‘GAL’	FRESH		81.78 ± 0.68^†^	−5.56 ± 1.32	19.59 ± 0.87
	FD	2.95 ± 2.45	79.81 ± 2.68	−4.85 ± 1.17	17.5 ± 1.58
	VMD 480	34.42 ± 4.2	54.16 ± 6.35	10.87 ± 1.73	31.93 ± 4.51
	VMD 120	27.7 ± 4.22	58.86 ± 4.8	1.49 ± 2.07	33.46 ± 1.43
	VMD 480/120	19.67 ± 1.82	64.48 ± 2.45f	−0.97 ± 1.34	27.75 ± 2.11
	CD-VMD 480/120	10.92 ± 2.84	72.4 ± 2.7	−1.71 ± 3.62	23.66 ± 1.42
	CD 50 °C	8.69 ± 4.51	75.21 ± 5.13	−0.95 ± 1.27	16.23 ± 1.59
	CD 60 °C	7.76 ± 3	74.43 ± 3.35	−3.2 ± 1.25	18.79 ± 1.48
	CD 70 °C	15.57 ± 8.45	67.37 ± 8.3	0.24 ± 2.39	18.45 ± 3.81
‘MSI’	FRESH	-	79.35 ± 1.49	−7.19 ± 3.03	21.82 ± 1.86
	FD	7.78 ± 3.9	71.86 ± 3.9	−6.34 ± 1.37	19.88 ± 1.05
	VMD 480	32.93 ± 13.78	49.98 ± 14.65	7.05 ± 5.94	26.19 ± 13.53
	VMD 120	20.1 ± 2.76	63.63 ± 2.2cd	−1.69 ± 1.43	33.08 ± 1.94
	VMD 480/120	17.13 ± 4.14	65.72 ± 4.34	−0.79 ± 2.52	29.99 ± 1.05
	CD-VMD 480/120	17.29 ± 2.9	63.18 ± 3.15	−1.42 ± 1.8	23.88 ± 1.81
	CD 50 °C	16.46 ± 2.26	64.62 ± 1.67	−0.84 ± 1.68	18.11 ± 3.01
	CD 60 °C	18.4 ± 3.81	62.8 ± 3.72	0.07 ± 1.43	18.38 ± 1.11
	CD 70 °C	17.5 ± 3.79	64.36 ± 4.68	1.38 ± 1.13	18.93 ± 0.72
‘PSI’	FRESH	-	78.88 ± 0.29	−6.19 ± 0.52	22.06 ± 0.6
	FD	4.38 ± 2.5	75.16 ± 3.53	−5.31 ± 0.92	19.92 ± 0.43
	VMD 480	18.42 ± 2.19	63.28 ± 1.95	0.09 ± 2.7	29.58 ± 1.81
	VMD 120	20.82 ± 1.09	62.15 ± 0.56	−0.31 ± 0.58	32.97 ± 2.58
	VMD 480/120	17.21 ± 2.53	64.23 ± 1.6	−1.04 ± 1.34	29.48 ± 2.19
	CD-VMD 480/120	12.46 ± 2.06	68.48 ± 2.2	−0.29 ± 2.36	25.57 ± 1.34
	CD 50 °C	13.45 ± 1.8	66.4 ± 1.5	−1.26 ± 1.39	21.06 ± 0.92
	CD 60 °C	12.8 ± 2.18	66.9 ± 1.88	−1.74 ± 2.23	22.93 ± 1.82
	CD 70 °C	13.82 ± 6.88	66.43 ± 6.83	−0.2 ± 1.77	21.62 ± 1.68
**Duncan’s Multiple Range Test**					
Drying treatment	FD	5.52c^†^	75.52e	−5.50	19.09a
	VMD 480	30.54e	54.89d	6.61d	28.20b
	VMD 120	23.11d	61.46c	−0.10ab	33.25d
	VMD 480/120	18.25b	64.73ac	−0.90ab	29.15b
	CD-VMD 480/120	13.91a	67.95ab	−1.12ab	24.37c
	CD 50	13.16a	68.66b	−1.00ab	18.45a
	CD 60	13.23a	67.96ab	−1.62a	20.06a
	CD 70	16.25a	65.67ab	0.57b	19.66a
Cultivar	‘GAL’	16.51ab	69.45a	−0.366a	23.07a
	‘MSI’	19.28b	64.47b	−0.795a	23.11a
	‘PSI’	14.44a	67.74a	−1.716a	25.08a

^†^ mean value followed by the same letter ± standard deviation SD values ^‡^ Values followed by the same letter, within the same column, are not significantly different (*p* < 0.05, Duncan’s multiple range test);—not detected; FD-freeze drying; CD-convective drying; VMD-vacuum-microwave drying; CD-VMD- convective-vacuum-microwave drying

**Table 3:** The influence of different methods and parameters on the total phenolic content (mg/100 g dm) and antioxidant capacity (ORAC, mmol TE/100 g dm) of jujube fruits.

Cultivars	Drying Conditions	Total Polyphenols Content	ORAC
‘GAL’	FD (control)	3424 ± 23^†^	48.56 ± 2.8
	CD 50 °C	3474 ± 44	38.79 ± 4.6
	CD 60 °C	2546 ± 16	37.51 ± 2.1
	CD 70 °C	1828 ± 29	32.99 ± 2.2
	VMD 120 W	2271 ± 31	54.54 ± 1.9
	VMD 480 W	2481 ± 18	44.59 ± 2.6
	VMD 480/120 W	2964 ± 25	45.09 ± 3.8
	CD-VMD	2881 ± 27	44.34 ± 1.1
‘MSI’	FD (control)	4287 ± 31	72.45 ± 3.5
	CD 50 °C	2377 ± 22	54.83 ± 2.7
	CD 60 °C	2481 ± 13	34.14 ± 2.2
	CD 70 °C	1555 ± 27	20.81 ± 1.8
	VMD 120 W	3237 ± 31	63.44 ± 3.1
	VMD 480 W	2791 ± 27	57.58 ± 2.1
	VMD 480/120 W	3920 ± 24	60.44 ± 3.3
	HaD-VMD	3438 ± 31	58.13 ± 1.4
‘PSI’	FD (control)	5870 ± 21	66.67 ± 2.7
	CD 50 °C	5696 ± 33	64.71 ± 1.9
	CD 60 °C	4493 ± 24	59.87 ± 2.5
	CD 70 °C	3458 ± 25	35.86 ± 3.1
	VMD 120 W	5343 ± 35	69.28 ± 3.8
	VMD 480 W	5244 ± 46	77.90 ± 4.1
	VMD 480/120 W	5076 ± 52	61.83 ± 2.6
	CD-VMD	4432 ± 14	48.91 ± 2.9
**Duncan’s Multiple Range Test**			
Drying treatment	FD (control)	4527a^‡^	62.56a
	CD 50 °C	3849ab	52.78abc
	CD 60 °C	3173bc	43.84c
	CD 70 °C	2280c	29.89d
	VMD 120 W	3617ab	62.42a
	VMD 480 W	3505ab	60.02ab
	VMD 480/120 W	3986ab	55.78ab
	HaD-VMD	3583ab	50.46bc
Cultivar	‘GAL’	2733b	43.30c
	‘MSI’	3010b	52.73b
	‘PSI’	4951a	60.63a

^†^ Values followed by the same letter ± standard deviation ^‡^ Mean values for each processing followed by different letters are statistically different at *p* < 0.05.
